# Levofloxacin-Based First-Line Therapy versus Standard First-Line Therapy for *Helicobacter pylori* Eradication: Meta-Analysis of Randomized Controlled Trials

**DOI:** 10.1371/journal.pone.0085620

**Published:** 2014-01-21

**Authors:** Musthafa Chalikandy Peedikayil, Fahad Ibrahim AlSohaibani, Abdullah Hamad Alkhenizan

**Affiliations:** 1 Department of Medicine, King Faisal Specialist Hospital & Research Center, Riyadh, Kingdom of Saudi Arabia; 2 Department of Family Medicine & Polyclinics, King Faisal Specialist Hospital & Research Center, Riyadh, Kingdom of Saudi Arabia; Cardiff University, United Kingdom

## Abstract

**Background:**

First-line levofloxacin-based treatments eradicate *Helicobacter pylori* with varying success. We examined the efficacy and safety of first-line levofloxacin-based treatment in comparison to standard first-line therapy for *H pylori* eradication.

**Materials and Methods:**

We searched literature databases from Medline, EMBASE, and the Cochrane Register of Randomized Controlled Trials through March 2013 for randomized controlled trials comparing first-line levofloxacin and standard therapy. We included randomized controlled trials conducted only on naïve *H pylori* infected patients in adults. A systematic review was conducted. Meta-analysis was performed with Review Manager 5.2. Treatment effect was determined by relative risk with a random or fixed model by the Mantel-Haenszel method.

**Results:**

Seven trials were identified with 888 patients receiving 7 days of first-line levofloxacin and 894 treated with standard therapy (Amoxicillin, Clarithromycin and proton pump inhibitor) for 7 days. The overall crude eradication rate in the Levofloxacin group was 79.05% versus 81.4% in the standard group (risk ratio 0.97; 95% CI; 0.93, 1.02). The overall dropout was 46 (5.2%) in the levofloxacin group and 52 (5.8%) for standard therapy. The dizziness was more common among group who took Levofloxacin based treatment and taste disturbance was more common among group who took standard therapy. Meta-analysis of overall adverse events were similar between the two groups with a relative risk of 1.06 (95% CI 0.72, 1.57).

**Conclusion:**

*Helicobacter pylori* eradication with 7 days of Levofloxacin-based first line therapy was safe and equal compared to 7 days of standard first-line therapy.

## Introduction

*Helicobacter pylori* infection is one of the most common chronic human bacterial infections. The consequences of infection are chronic gastritis, gastric and duodenal ulcers, gastric cancer, and primary gastric lymphoma of mucosa-associated lymphoid tissue type (MALT lymphoma) [1]. Eradication of *H pylori* may cure dyspepsia [2], peptic ulcer disease, and MALT lymphoma [3].

Various consensus groups, including the group at the Maastricht consensus meeting [2], recommended a triple-therapy regimen containing a proton pump inhibitor (PPI), clarithromycin, and either amoxicillin or metronidazole as first-line treatment for the eradication of *H pylori* [2]. However, recent reports indicate that these regimens achieve only about a 70% eradication rate [4]–[5]. The most important cause for the reduced success of standard triple therapy is the increasing rate of *H pylori* clarithromycin/metronidazole resistance [6].

There have not been any new drugs recently developed to treat *H pylori*. Thus, many investigators have examined various combinations of antibiotics or sequential therapies to improve the eradication rate. Many studies have investigated levofloxacin-based regimens as second-line treatments, including meta-analyses [7]. The 2012 Maastricht consensus guideline recommends the levofloxacin-PPI containing regimen for patients who have failed standard first-line treatment [2]. Many studies have examined levofloxacin-PPI as a first-line therapy for eradication of *H pylori* infection [8]–[10]. However, the eradication rates achieved with first-line levofloxacin-based treatments are not uniform. Some reports show improved efficacy with levofloxacin-based regimens in comparison to standard first-line therapy, whereas others have found equivalent or poorer responses [9], [11]–[12].

Conclusive data regarding the role of levofloxacin-based first-line therapy for *H pylori* eradication does not currently exist. A meta-analysis may provide meaningful answers to this question. Thus, a systematic review and meta-analysis was conducted. First objective was to do a meta-analysis of data from all of the available randomized, controlled trials that compared 7 days of Levofloxacin based treatment to 7 days of Amoxicillin, Clarithromycin and PPI as first line treatment for *H pylori* eradication. The second objective was to perform a meta-analysis of adverse effects of levofloxacin-based first-line therapy versus standard first-line therapy.

## Materials and Methods

### Selection of studies

A particular trial was included in the meta-analysis if it satisfied following. (1) The study must have been a randomized, controlled trial. (2) The presence of *H pylori* infection before and after treatment must be confirmed by one or more diagnostic methods. (3) The subjects must have been at least 18 years old. (4) The patients were naïve to *H pylori* eradication treatment. (5) One group was treated with standard first-line therapy containing a minimum of two antibiotics and one PPI (amoxicillin, clarithromycin, with a PPI). (6) The levofloxacin-based treatment group was treated with a minimum of two antibiotics, including levofloxacin, and one PPI. Randomized, controlled trials (RCTs) with more than two treatment groups were selected if two of the treatment groups satisfied the inclusion and exclusion criteria. Studies or articles were excluded if sequential therapy was the only treatment regimen, levofloxacin was included in all of the treatment groups, studies included known antibiotic susceptibility tests, manuscripts were review articles, *H pylori* eradication therapy was provided for treatment failures, or if data were incomplete.

### Literature search

Medline, EMBASE, and the Cochrane Register of Randomized Controlled Trials were searched for relevant studies. In addition, a manual search was performed on the references of relevant published articles. The literature search was performed without any restrictions on language. The Medline search was performed with the keywords levofloxacin and *Helicobacter pylori* and included articles from 1956–March 2013. The Cochrane library was searched for articles with the keywords levofloxacin, *Helicobacter pylori*, and randomization. Each title and abstract was read, and potential articles were recorded on a short list. All of the articles on the short list were downloaded or purchased. EndNote version X3 (Thomson Reuters) was used at various stages for collecting, storing, and finding duplicate references. Duplicates were identified and removed.

### Data extraction

Each researcher independently collected data in a predefined data extraction form. Any difference of opinion between researchers was resolved to reach a consensus. The following data were collected from each paper: year of publication, institution, study design, quality of the study, tests performed to confirm the diagnosis and eradication, gender, age, medications in each arm, drop outs, and adverse events. Additional data regarding medications were collected and included the drug name, dose, frequency of treatment, and duration of treatment. Eradication rates expressed in the trials as intention to treat analysis were used.

### Quality assessment

Each investigator independently assessed the quality of the studies by using the Jadad-Score. This score was entered into the predefined data extraction form. The following quality indicators were assessed: randomization (generation of allocation sequence), allocation of concealment, blinding, description of dropouts, and description of loss of follow-up. The maximum score was five. A higher score represented a higher quality paper. In this meta-analysis, studies with scores of three and above were included in the final meta-analysis.

### Statistical analysis

Data were independently extracted by the same reviewer and cross-checked. Discrepancies were resolved by discussion. Data synthesis was performed with the Cochrane Statistics package RevMan version 5.2. Relative risk (RR) and risk difference (RD) with 95% confidence intervals (CI) were reported. If there was a statistically significant RR, then the number needed to treat (NNT) was calculated. Heterogeneity was tested by the *I^2^* statistics method. A value greater than 50% indicated substantial heterogeneity. Potential causes of heterogeneous treatment effects were explored by pre-specified subgroup analysis. If statistically significant heterogeneity existed between studies, the weighted estimate of the typical treatment effect across trials (relative risk) was calculated with the random effects model to ensure robust results.

## Results

### Description of selected studies

**Figure 1:**
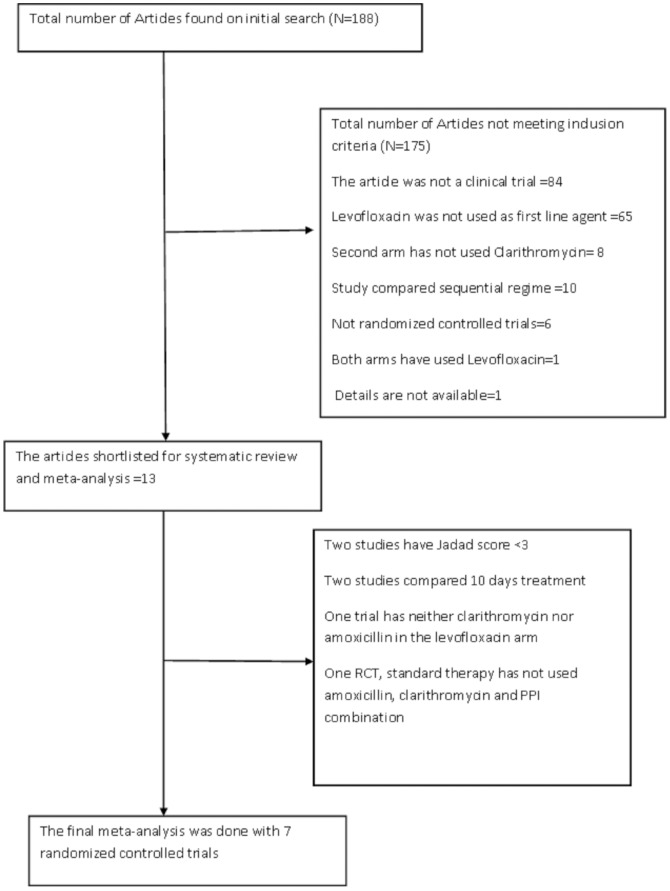
Flow chart showing study selection.

The literature search was performed with predefined keywords through different search engines as described in the methods section. Our initial search revealed a total of 188 articles. Each abstract was reviewed to find suitable RCTs that satisfied the inclusion and exclusion criteria. Figure 1 shows the flow diagram of the process by which articles were selected. Finally, 13 RCTs met our predefined inclusion and exclusion criteria and were qualified for the meta-analysis [8]–[9], [11]–[21]. From 13 RCTs, two of them [16], [20] were excluded because of their Jadad score was <3, three of them [8], [11], [14], compared 10 days of levofloxacin based regimen to 10 days of standard therapy, and the study by Zullo et al [21] was excluded because the standard arm was unique and contained 7 days of Clarithromycin, Tinidazole, Bovine Lactoferrin and Rabeprazole. The quality of each RCT was based on the Jadad score (Table 1).

**Table 1:** Quality assessment of selected studies.

Author	Randomization	Concealment of allocation	Blinding	Adequacy of blinding	Drop out described	Jadad score
**Assem M**	Yes	Yes	Open label	N D	Yes	3
**Chen LW**	Yes	Yes	Open label	N D	Yes	3
**Choi KH**	Yes	Yes	Double Blind	Adequate	Yes	5
**Hung IF**	Yes	Yes	Open label	N D	Yes	3
**Iacopini F**	Yes	Yes	Open label	N D	Yes	3
**Liou JM**	Yes	Yes	N D	N D	Yes	3
**Nista EC**	Yes	Yes	Open label	N D	Yes	3

ND = Not described.

Several articles reporting results for levofloxacin-based first-line therapy for *H pylori* eradication were excluded. The RCTs by Cammarota et al. and Di Caro et al. were excluded, because all of the treatment groups included levofloxacin [22], [23]. The RCT by Antos et al. was excluded, because the study included patients with one or more previous treatment failures [24]. The study by Schrauwen et al. was excluded, because it was not a randomized, controlled trial, and both groups were treated with levofloxacin. Finally, the study by Castro-Fernandez et al. was excluded, because the study was not a randomized, controlled trial [25].

The cumulative number of *H pylori*-infected patients enrolled in the 7 studies included in our meta-analysis was 1782, which included 888 who received levofloxacin-based first-line therapy and 894 treated with standard first-line therapy. The selected studies were from different countries. Two of the selected RCTs were from Italy; two studies were from Taiwan, one each from Hong King, Korea and one from Middle East (Saudi Arabia and Egypt).

All of the patients were older than 18 years. Further, none of the patients had received prior treatment for *H pylori* infection. All of the RCTs confirmed the diagnosis of *H pylori* infection by upper endoscopy with gastric biopsy and histology except for one study, which confirmed the diagnosis by Urea breath test. All researchers have confirmed Eradication of *H pylori* infection a urea breath test.

Five of the 7 RCTs included the combination of levofloxacin, amoxicillin, and a PPI as first-line therapy (levofloxacin arm) and two RCTs included levofloxacin, clarithromycin, and a PPI. All seven RCTs included standard therapy with amoxicillin, clarithromycin, and a PPI. Four studies had two treatment arms and other three RCTs had 3 treatment arms. Table 2 describes treatment effects, common adverse events, and drop outs of seven trials which are included in the meta-analysis.

**Table 2:** Details of treatment effects, common adverse events, and drop outs for studies included in the meta-analysis.

Author	Medications	Duration of Rx (N)	Eradication (N)	Recruitment (N)	Eradication rate ITT (%)	Drop out (N)	Adverse events (N)	Adverse events	NNT	Relative risk reduction (95% CI)
Assem M [13] LEVO arm	AMOX 1 gm. BID; LEVO 500 mg BID; ESOM 20 mg BID.	7	127	150	84.7	4	120	Nausea, Diarrhea, Taste disturbance		
Assem M [13] STD Arm	AMOX 1 gm BID; CLAR 500 mg BID; ESOM 200 mg BID	7	118	150	78.6	5	132	Nausea, Diarrhea, Taste disturbance	16.7	7.1 (−3.5 to 16)
Chen LW [15] LEVO arm	CLAR 500 mg QD; LEVO 500 mg QD; ESO 40 mg QD	7	71	90	78.9	5	8	Taste disturbance		
Chen LW [15] STD arm	AMOX 1 gm BID; CLAR 500 mg BID; ESO 40 mg BID.	7	74	99	74.8	13	12	Taste disturbance, Skin rash	24.1	5.2 (−10.8 to 19)
Choi KH [17] LEVO arm	AMOX 1 gm BID; LEVO 200 mg BID; OMEP 20 mg BID.	7	64	98	65.3	11	72	Diarrhea, anorexia, nausea		
Choi KH [17] STD arm	AMOX 1 gm BID; CLAR 500 mg BID; OMEP 20 mg BID	7	77	99	77.8	9	111	Diarrhea, anorexia, nausea, taste disturbance	−8	−19 (−42 to 0.4)
Hung IF [9] LEVO arm	AMOX 1 gm BID; LEVO 500 mg QD; ESOM 20 BID.	7	128	150	85.3	0	31	Dizziness, diarrhea		
Hung IF [9] STD arm	AMOX 1 gm BID; CLAR 500 mg BID; ESOM 20 mg BID.	7	139	150	92.7	0	43	Diarrhea, taste disturbance	−13.6	−8.6 (−17.7 to −0.2)
Iacopini F [12] LEVO arm	AZIT 500 mg QD; LEVO 500 mg QD; ESOM 20 mg QD.	7	54	83	65	6	11	Taste disturbance, diarrhea		
Iacopini F [12] STD arm	AMOX 1 gm BID; CLAR 500 mg BID; ESOM 20 mg BID.	7	53	81	65	11	35	Taste disturbance, nausea, diarrhea	−2.68	−0.6 (−25.7 to 20)
Liou JM [18] LEVO arm	AMOX 1 gm BID; LEVO 750 mg QD; LANS 30 mg BID.	7	161	217	74.2	16	163	Diarrhea, dizziness, taste disturbance		
Liou JM [18] STD arm	AMOX 1 gm BID; CLAR 500 mg BID; LANS 30 mg BID.	7	180	215	83.7	9	128	Diarrhea, taste disturbance,	−10.5	−12.8 (−24.5 to −2.3)
Nista EC [19] LEVO arm	CLAR 500 mg BID; LEVO 500 mg QD; ESOM 20 mg BID.	7	87	100	87	4	51	Taste disturbance		
Nista EC [19] STD arm	AMOX 1 gm BID; CLAR 500 mg BID; ESOM 20 mg BID.	7	75	100	75	5	38	Diarrhea	8.3	13.8 (1.2 to 25)

Abbreviations

AMOX = Amoxicillin; LEVO = Levofloxacin; ESOM = Esomeprazole; CLAR = Clarithromycin; OMEP = Omeprazole; LANS = Lansoprazole; AZIT = Azithromycin.

Rx = treatment; BID = twice daily; QD = once daily; N = number; NNT = number needed to treat; ITT = intention to treat; LEVO arm = levofloxacin arm; STD arm = standard arm.

gm = gram; mg = milligram.

**Figure 2:**
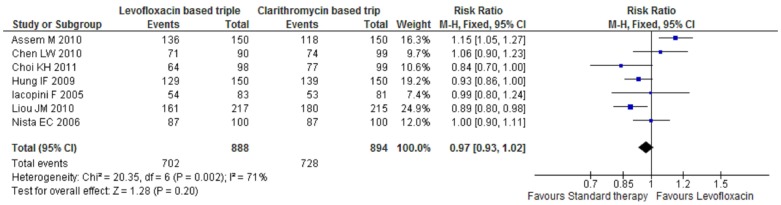
Forest plot showing the treatment effect of 7 randomized controlled trials. The crude eradication rate was similar between Levofloxacin based first line treatment and standard first line treatment for the eradication of *H pylori*.

Meta-analysis of seven trail's crude eradication rate was 79.05% versus 81.4% in the standard group and 79.5% in the levofloxacin-based first-line therapy. The difference in the crude eradication was not statistically significant with a relative risk of risk ratio of 0.97; 95% CI; 0.93, 1.02. Figure 2 shows the Forest plot of meta-analysis of 7 RCTs.

The heterogeneity of treatment effects in the meta-analysis was significant. Statistical analysis indicated that the *I^2^* statistics for the heterogeneity of the meta-analysis of seven RCTs were 71%.

**Figure 3:**
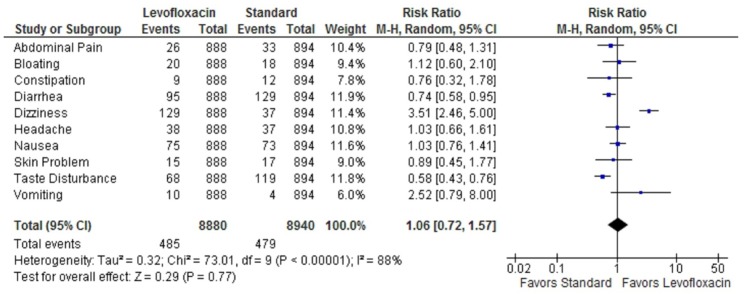
The Forest plot showing meta-analysis of common side-effects related to levofloxacin-based first line therapy, and standard first-line therapy. Overall side effects were equal for both levofloxacin and standard treatment.

The overall dropout was 46 (5.2%) in the levofloxacin group and 52 (5.8%) for standard therapy. The dizziness was more common among group who took Levofloxacin based treatment, and taste disturbance was more common among group who took standard therapy. Meta-analysis of overall adverse events were similar between the two groups with a relative risk of 1.06 (95% CI 0.72, 1.57). Figure 3 shows the Forest plot for the results of meta-analysis side effect with both treatment regimens.

**Figure 4:**
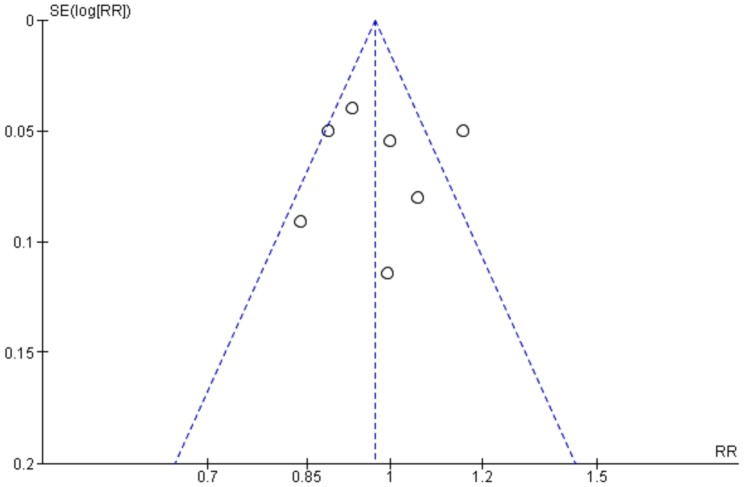
Funnel plot of included studies showing symmetrical distribution, and no publication bias.

The funnel plot (figure 4) of all seven included studies showed a symmetric pattern suggested that there are no publication biases.

## Discussion

The most significant finding of this meta-analysis was that levofloxacin-based first-line therapy and standard therapy for previously untreated *H pylori*-infected patients achieved equivalent outcomes. The overall eradication rates of the seven RCTs were 79.05% for levofloxacin-based regimens and 81.4% for standard therapy. The overall adverse events were similar between these two regimens.

Significant heterogeneity was observed in our meta-analysis. The obvious reasons for high heterogeneity were not evident. The main difference in the studies was in the study population. Otherwise duration of the treatment and medications used in the levofloxacin based first line therapy and standard therapy was more or less similar.

Since the year 2000, many investigators have published individual studies examining treatment of *H. pylori* with levofloxacin-based first-line therapy. The current meta-analysis included only randomized, controlled trials with predefined inclusion and exclusion criteria. Hence, we were forced to exclude many excellent studies from our meta-analysis. The *H. pylori* eradication rates in the excluded studies ranged from 75% to 96% with levofloxacin-based first-line therapy [22]–[23], [25]–[26]. The results of these individual studies support the concept that the efficacy and safety of levofloxacin-based first-line therapy is equivalent to standard first-line therapy.

Prior to this meta-analysis, another meta-analysis was performed, in which Zhang et al [27] compared levofloxacin-based first-line therapy to standard therapy. The previous meta-analysis found that a PPI and levofloxacin-based triple therapy was more effective than standard triple therapy with a lower rate of adverse events compared to standard triple therapy. That article was published in the Chinese language in 2009, and only the abstract was available in the English language. Therefore, the details of the RCTs that were included in that meta-analysis were not available to us.

The recently updated guidelines by the Maastricht IV/Florence Consensus Report [2], the American College of Gastroenterology Guideline on the management of *H pylori* [28], and Second Asia–Pacific Consensus Guidelines for *H pylori* infection [29] recommend levofloxacin-based triple therapy as a second-line treatment for *H pylori* infections. Two meta-analyses support these recommendations. The levofloxacin-PPI-based regimen was successful in 81% of patients with previous eradication failures compared to 70% of patients who responded to quadruple therapy [30]. The second meta-analysis reviewed the levofloxacin-based triple salvage therapy (levofloxacin+amoxicillin+PPI), which was found to be superior to bismuth-based quadruple salvage therapy [31].

Should we recommend levofloxacin-based regimens as first-line therapies for *H pylori* eradication? Our current meta-analysis suggests that levofloxacin-based first-line treatment is as effective as standard therapy for *H pylori* eradication, and the adverse events and dropout rates are also equal. Noticeably, the overall eradication rates for *H pylori* with both levofloxacin-based regimens and standard therapy were around 80%.

The second reason for not recommending levofloxacin-based therapy as a first-line treatment modality for *H pylori* infection is the increasing incidence of levofloxacin-resistant *H pylori* strains from different parts of the world, especially countries in which quinolone consumption is high. Levofloxacin-resistant *H pylori* has been reported in the range of non-existent in Malaysia to 30.3% in China [32]–[33]. Levofloxacin resistance has been reported to be 11.7% [95% CI 6.5–20.3%)] in Ireland with much higher rates of resistance in subjects older than 45 years (19.1%) in comparison to those younger than 45 years (2.6%) [34]. *H pylori* resistance to levofloxacin was 5% in Poland and 5.3% in Iran [6], [35]. An Italian study by Zullo et al. reported a levofloxacin resistance rate of 19.1% with higher resistance rates in older patients compared to younger patients (28.4% vs. 14.4%, P = 0.048) [36]. In Taiwan, the prevalence of levofloxacin resistance in *H pylori* isolates has increased from 3.2% in isolates collected before 2004 to 16.3% in isolates collected after 2004 [37].

Various studies indicate that resistance will reduce the efficacy of levofloxacin-based regimens in the eradication of *H pylori*. In Italy, the eradication rate decreased significantly from 75% in levofloxacin-susceptible patients to 33.3% in patients with levofloxacin-resistant strains [38]. A study from the U.S.A. suggests that levofloxacin resistance may be associated with prior quinolone exposure during the past 10 years [39].

There are some suggestions that levofloxacin-based first-line therapy may be used in areas where clarithromycin resistance is higher than 20%, along with levofloxacin resistance is below 10%. For patients who are allergic to penicillin, instead of Metronidazole-containing regimens, the levofloxacin based regime can be used as a first line therapy [40]–[41]. However, these regimens have not yet been compared in the first-line setting. Thus, further studies are needed to test this concept.

Although there are a few limitations to this meta-analysis, such as heterogeneity, there are numerous strengths. Levofloxacin-based first-line therapy has been included in clinical practice since the year 2000. However, a thorough meta-analysis investigating the role of levofloxacin in the eradication of *H pylori* has been lacking. Our meta-analysis was successful in identifying all of the important randomized, controlled trials with strict criteria and provided clear answers to various questions. To our knowledge, no other meta-analysis comparing first-line levofloxacin-based therapy to first-line standard therapy has been published in the English language.

## Conclusions

This meta-analysis suggests that levofloxacin-based first-line therapy and standard therapy have equivalent efficacy and safety profiles for eradication of *H pylori*. There is not sufficient evidence to support replacement of the current standard first-line therapy with levofloxacin-based first-line therapy. Levofloxacin-based first-line therapy may be a valuable alternative for patients with penicillin allergies and in areas in which quinolone resistance is very low and clarithromycin resistance is very high. Each country should determine the prevalence of antibiotic resistance within their community before introducing new treatment regimens for *H pylori*.

## Supporting Information

Table S1 **Prisma checklist.**
(DOC)
